# Geographic differences in the magnitude of black‐white disparities in having obesity

**DOI:** 10.1002/osp4.679

**Published:** 2023-05-26

**Authors:** Steven A. Cohen, Monique J. Brown, Furong Xu, Caitlin C. Nash, Mary L. Greaney

**Affiliations:** ^1^ Department of Health Studies University of Rhode Island Kingston Rhode Island USA; ^2^ Department of Epidemiology and Biostatistics Arnold School of Public Health University of South Carolina Columbia South Carolina USA; ^3^ School of Education Alan Shawn Feinstein College of Education and Professional Studies University of Rhode Island Chafee Social Science Center Kingston Rhode Island USA

**Keywords:** geographic factors, health disparities, obesity, rural‐urban status, social determinants

## Abstract

**Background:**

Obesity disparities in the United States are well documented, but the limited body of research suggests that geographic factors may alter the magnitude of these disparities. A growing body of evidence has identified a “rural mortality penalty” where morbidity and mortality rates are higher in rural than urban areas, even after controlling for other factors. Black‐White differences in health and mortality are more pronounced in rural areas than in urban areas.

**Objective:**

Therefore, the purpose of this study was to explore how rural‐urban status and region moderate Black‐White health disparities in obesity.

**Methods:**

Data were abstracted from the 2012 Behavioral Risk Factor Surveillance System, with the sample being restricted to Black and White respondents (n = 403,231). Respondents’ county of residence was linked to US Census information to obtain the county‐level Index of Relative Rurality (IRR) and Census division. Crude and adjusted logistic regression models were utilized to assess the magnitude of Black‐White disparities in having obesity (yes/no) by IRR quartile and by Census division.

**Results:**

Overall, Black‐White differences in obesity were wider in rural than in urban counties, with a significant linear trend (p < 0.001). Furthermore, when stratified by US Census division, results revealed that disparities were significantly wider in rural than urban areas for respondents living in the Middle Atlantic and South Atlantic divisions. In contrast, the association was reversed for the remaining divisions (New England, East North Central, West North Central, Mountain, and Pacific), where the magnitude of the Black‐White difference was the largest in urban areas.

**Conclusion:**

Findings highlight the need to understand and account for critical place‐based factors that exacerbate racial obesity disparities to develop and maximize the effectiveness of policies and programs designed to reduce racial inequalities and improve population health.

## Graphical abstract

Our study aims to explore how rural‐urban status and the US region moderate Black‐White differences in obesity. Black‐White differences in obesity were wider in rural than urban counties, with a significant linear trend (*p* < 0.001). When stratified by Census division, the magnitude and direction of those rural‐urban differences varied by geographic location, which is important to recognize when crafting policies and programs designed to reduce disparities in obesity.

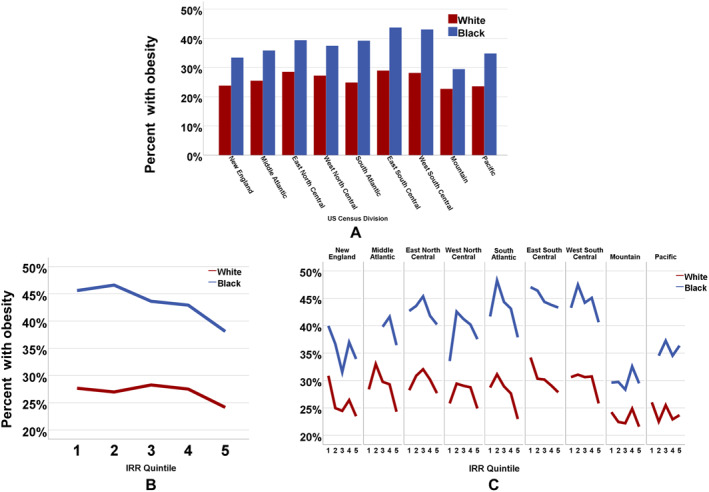

## 1 BACKGROUND

Social determinants of health (SDH)—the social, economic, and environmental factors that shape health and can be influenced by social policies and conditions^1^—are a critical driver of health disparities. There are well‐documented disparities from SDH in obesity by race and ethnicity.^2^, ^3^, ^4^, ^5^, ^6^, ^7^, ^8^, ^9^ For example, a 2018 study found that the prevalence of obesity in the US was 44% higher among Black women than non‐Hispanic White women, and 8% higher among Black men than non‐Hispanic White men.^10^ The causes of these racial disparities are complex and can occur at the individual, interpersonal, community, environmental, and policy levels, that evolve throughout the lifespan, according to the Social Ecological Model of Health.^11^ Multiple individual factors likely contribute to these Black‐White differences in having obesity at the individual level. Extensive research has assessed the contributions of physical activity^9^, ^12^, ^13^ and nutrition‐related behaviors^14^ to Black‐White differences in obesity. A decomposition analysis^15^ of both physical activity and nutritional attainment aligns with previous research^16^, ^17^, ^18^, ^19^, ^20^, ^21^ showing that diet quality likely contributes more to these differences in having obesity than a lack of physical activity, although both are important factors. Other research has suggested that, in addition to nutrition and physical activity, chronic stress,^22^ socioeconomic status,^9^, ^23^, ^24^ and educational attainment^25^, ^26^ also play critical roles in promoting Black‐White differences in having obesity.

Structural and environmental factors, such as structural racism, impact individual health behaviors through a variety of mechanisms, which, in turn, can modulate the risk of obesity across the lifespan.^27^, ^28^, ^29^ For example, de facto racial segregation is one way in which structural racism manifests. Segregation contributes to differential opportunities for employment, education quality and access, and homeownership.^30^ It can also create obesogenic environments and influence individual behaviors through the implementation of policies, planning, and zoning that can limit the availability of healthy food options and opportunities for exercise and physical activity due to a lack of investment and safety concerns.^31^ In addition to residential segregation, systemic racism impacts health behaviors that lead to higher levels of obesity and other adverse health outcomes through other pathways. These include but are not limited to, unfair lending practices and related barriers to home ownership and taxation, biased policing, and voter suppression.^32^ Measuring how these manifestations of structural racism impact health and health behaviors is challenging, but important, and deserves further attention. That said, one recurring, plausible mechanism through which structural racism impacts health is the sustained physical toll of concentrated and psychosocial stressors across the lifespan,^33^ which both directly impacts health, as well as individual health behaviors that can promote obesity.

In addition to those factors mentioned above, the overall magnitude of Black‐White differences in obesity prevalence also varies substantially by geography.^34^ There is increasing recognition that place‐based factors, such as neighborhood, community, and state characteristics, contribute to obesity rates and Black‐White differences in obesity rates. These factors include, but are not limited to, neighborhood disadvantage,^35^ segregation,^36^, ^37^ and access to healthy foods.^38^ For example, a study of a well‐integrated, low‐income neighborhood found that racial disparities are reduced somewhat when community‐level socioeconomic factors, such as income and education, are equalized.^39^

More broadly, other research suggests that policies, even those not directly aimed at affecting health outcomes, likely contribute to structural racism and racial disparities in obesity.^40^ One such example is zoning policies, which influence where business can be built in a community. Such policies can impact the availability of food, green space, and other services impact obesity.^40^, ^41^ Additionally, state and community tax policies can alter the relative costs and ease of obtaining healthful versus unhealthful foods, with the availability of healthful foods more likely in more affluent, White neighborhoods.^42^ These zoning policies, land use regulations, and other financial incentives have been the focal point of considerable discussion in addressing the systematic causes of Black‐White differences in obesity and other population health measures.^43^

Another community attribute, rural‐urban status, has also been shown to influence population health outcomes. Studies have identified a “rural mortality penalty”,^44^ where mortality rates are higher in rural than urban areas of the US, even after controlling for other factors, including SDH.^45^ This rural mortality penalty extends to other health outcomes, including preventive health behaviors,^46^ COVID‐19 outcomes,^47^ cancer screenings^48^ and drug overdoses.^49^ However, the driving forces behind the observed rural mortality penalty are not well understood. Fundamental cause theory suggests that rural areas may be more likely to provide or promote the underlying social conditions that give rise to more proximal causes of death and morbidity such as high poverty, unemployment, and lower education.^50^ Although rural areas tend to have higher levels of green space and better air and water quality, they may be less likely to have a built environment conducive to physical activity and recreation.^51^ Cultural aspects of rural areas may also play a role. For example, food preparation methods and celebrations and events revolving around unhealthy foods are more common in rural areas of the US.^52^, ^53^ Furthermore, evidence suggests that people living with obesity living in rural areas are more likely to perceive themselves as healthy and adopt fatalistic beliefs about weight and health compared to those living in non‐rural areas.^54^ However, the individual contributions of these and other attributes of rural environments to the rural mortality penalty are unknown and deserve further study.

There is growing evidence that this “rural mortality penalty” not only contributes to higher levels of premature mortality and morbidity in rural areas but may also exacerbate other types of health problems. Recent studies have shown that Black‐White differences in health and mortality are more pronounced in rural areas than urban areas. Black‐White differences in mortality are widest in rural areas, and those differences have widened over time.^55^ Similar results have been found with obesity: rural areas have the highest rates of obesity, but also Black‐White differences in the rates of having obesity are largest in rural areas.^56^ Furthermore, a 2021 study^57^ found critical regional differences in the rural health and mortality penalty in the US, where rural mortality rates are worse in the rural south compared to other rural areas. However, to date, no research has examined how Black‐White differences in obesity vary by both rural‐urban status and US region. Therefore, the objective of this study was to examine regional differences in how the magnitude of Black‐White differences in obesity vary by rural‐urban status using a large, nationally representative sample of US adults. We hypothesized that the magnitude of Black‐White differences would be greatest in rural areas, particularly in the South and Midwest.

## 2 METHODS

### 2.1 Data

This study is a secondary data analysis of the 2012 Behavioral Risk Factor Surveillance System (BRFSS), the largest network of health‐related telephone surveys administered by the Centers for Disease Control and Prevention. The BRFSS collects data from US community dwelling residents 18+ years of age in all 50 states, as well as Guam and Puerto Rico, regarding their demographics, self‐reported health‐related behaviors, use of preventive services, and other health‐related information, and guides planning and prevention efforts at the state and federal levels.^58^ Over 400,000 interviews with BRFSS respondents aged 18 and older are conducted annually.

This study used the 2012 BRFSS sample, the most recent year in which the respondent's place of residence (county) was collected. The 2012 BRFSS included 475,687 total respondents, with response rates of 49.1% and 35.3% for landlines and cell phones, respectively.^59^ The analytic sample for the current study was restricted to respondents who provided information on height and weight and all other key study variables that were living in the contiguous US (lower 48 states), and who responded “White” or “Black” as their preferred race. The county of residence was not available in the 2012 BRFSS for residents of Alaska and Hawaii. The resultant sample size was 403,231 (84.8%). Each respondent was linked to area‐level data from the 2010 US Census^60^ and American Community Survey 5‐year estimates^61^ via county Federal Information Processing Standard code.

### 2.2 Measures

#### 2.2.1 Outcome variable

The main outcome variable of interest was obesity (yes vs. no). Obesity status was determined by BMI, which was calculated using self‐reported height and weight. Respondents with a BMI of 30 kg/m^2^ or above were classified as having obesity.^62^

#### 2.2.2 Predictor variables

The main predictor variables were race, US Census division, and rural‐urban status. Race was obtained and categorized in several ways in the BRFSS data. Respondents were asked “Which of these groups best represents your race?” Possible responses were “White”, “Black or African American”, “Asian”, “Native Hawaiian or Other Pacific Islander”, or American Indian/Alaska Native”. The analytical sample was restricted to respondents reporting their race as being either “Black or African American” or “White”.

The US Census division for each respondent was obtained through the state of residence. The four US Census regions (Northeast, South, Mid‐West, and West) were categorized into nine US Census divisions: New England, Mid‐Atlantic, East North Central, West North Central, South Atlantic, East South Central, West South Central, Mountain, and Pacific. For a list of states in each US Census division, see Supplemental Table S1 (adapted from^63^).

Rural‐urban status was obtained through the Index of Relative Rurality (IRR), a continuous, composite measure of four measures of rural‐urban characteristics—population size, population density, percent urban population, and distance to nearest metropolitan area.^64^, ^65^ The IRR has been used in prior research on rural‐urban health inequities.^66^, ^67^, ^68^, ^69^, ^70^, ^71^ For analysis, all US counties were categorized into IRR quintiles with a quintile of 1 being the most rural and a quintile of 5 being the most urban.^70^, ^71^

#### 2.2.3 Covariates and complex sampling

Other variables of interest included in this analysis were respondents' age (in years), sex (female, male), annual income category (<$25,000, $25,000–49,999, ≥$50,000, missing/not available), currently employed for pay (yes, no), education (bachelor's degree or higher vs. less than bachelor's degree), currently married (yes, no), and current smoker (yes, no). The BRFSS data set included the Centers for Disease Control and Prevention's analytic sample weights, which were used in all analyses to account for differences in sampling and response probabilities, in accordance with BRFSS guidelines.^59^

### 2.3 Data analysis

Descriptive statistics were obtained for all study variables, including weighted means and standard deviations or medians and interquartile ranges for all continuous and discrete variables, and weighted frequencies and percentages for all categorical variables. Bivariate associations were obtained through the use of chi squared tests, *t*‐tests, Wilcoxon rank sum tests, and Pearson and Spearman correlation coefficients, depending on the variable. Differences in the distribution of respondents by US Census division and rural‐urban status (IRR quintile) were explored using crosstabulations and descriptive graphs. Black‐White differences were tabulated across all study variables, including the outcome of interest (having obesity) and geographic indicators (US Census division and IRR quintile).

Weighted generalized linear models with a logistic link function were used to assess the magnitude of the difference between Black and White respondents with respect to obesity by US Census division and IRR quintile. Four sets of models were obtained. First, Black‐White differences were examined in the entire sample, both unadjusted and including covariates (age, sex, income, employment, marital status, and smoking status). Second, Black‐White differences were stratified by IRR quintile and then by US Census division. Third, Black‐White differences were then modeled for each IRR quintile‐US Census division category. For the second and third sets of models, obesity (yes/no) was modeled both unadjusted and adjusted for covariates for which a set of propensity scores were initially created based on the covariates using the complete data set. These propensity scores were used to address confounding without using excessive degrees of freedom using the covariates above as predictors (age, sex, income, employment status, marital status, and smoking status). Respondents were then ranked according to their estimated propensity score and were stratified into subsets based on decile of the propensity score for the analysis in these models.^72^

Lastly, interaction terms were incorporated for each US Census division to assess the potential for monotonic trends in the association between the IRR quintile and the magnitude of the association comparing Blacks to Whites in having obesity using race*IRR interaction terms. For all models, the model fit was evaluated using pseudo‐R‐squared and Akaike Information Criteria statistics. SAS version 9.4 (Cary, NC) and IBM SPSS version 28 (Armonk, NY) were used to analyze the data. Statistical significance was set at *p* < 0.05 for all analyses.

## 3 RESULTS

Descriptive statistics for the sample overall and by race (Black vs. White) are shown in Table 1. Just over one‐quarter of the sample (27.0%) had obesity, with 25.6% of White respondents and 38.4% of Black respondents having obesity. The mean age of the analytic sample was 56.3 years, and most of the sample was female (60.2%), employed for pay (50.5%), had less than a bachelor's degree (64.3%), married (53.4%), and were non‐smokers (83.8%). Just under half of the White respondents (41.4%) reported having an income of at least $50,000, compared to 21.2% of the Black respondents. The majority of respondents (57.2%), both Black (75.2%) and White (55.7%), resided in the most urban quintile (quintile 5, Q5) of US counties. The US Census divisions contributing the most respondents to the sample were the South Atlantic (16.2%), the West North Central (16.0%), and the Mountain (13.3%) divisions. All associations comparing Black and White respondents were statistically significant (*p* < 0.001).

**TABLE 1:** Frequencies [*N* (weighted %)] for analytic sample from the Behavioral Risk Factor Surveillance System (BRFSS), 2012.

		Overall	White	Black
Age	Mean (SD)	56.3 (17.3)	56.8 (17.2)	52.3 (17.0)
Sex	Female	242587 (60.2)	215816 (59.4)	26771 (66.8)
	Male	160644 (39.8)	147323 (40.6)	13321 (33.2)
Income ($)	<25,000	98237 (24.4)	81103 (22.3)	17134 (42.7)
	25,000–49,999	92249 (22.9)	83248 (22.9)	9001 (22.5)
	50,000+	157774 (39.1)	149270 (41.1)	8504 (21.2)
	Missing	54971 (13.6)	49518 (13.6)	5453 (13.6)
Currently employed for pay	Yes	198718 (49.5)	180741 (50.0)	17977 (45.2)
	No	202731 (50.5)	180912 (50.0)	21819 (54.8)
Bachelor's degree or higher	Yes	143333 (35.7)	133785 (36.9)	9548 (23.9)
	No	258683 (64.3)	228325 (63.1)	30358 (76.1)
Married	Yes	214151 (53.4)	201966 (55.9)	12185 (30.6)
	No	187150 (46.6)	159538 (44.1)	27612 (69.4)
Current smoker	Yes	64078 (16.2)	56605 (15.9)	7473 (19.2)
	No	331529 (83.8)	300078 (84.1)	31451 (80.8)
Has obesity	Yes	108754 (27.0)	92965 (25.6)	15789 (38.4)
	No	294477 (73.0)	270174 (74.4)	24303 (60.6)
General health status	Fair or poor	74567 (18.5)	63569 (17.6)	10998 (27.5)
	Good, very good, or excellent	327447 (81.5)	298511 (82.4)	28936 (72.5)
Index of relative rurality quintile	1 (most rural)	9843 (2.7)	9264 (2.9)	579 (1.6)
	2	31852 (8.9)	30322 (9.4)	1530 (4.1)
	3	43408 (12.1)	40481 (12.6)	2927 (7.9)
	4	66769 (18.6)	62664 (19.4)	4105 (11.1)
	5 (most urban)	207285 (57.7)	179535 (55.7)	27750 (75.2)
US Census division	New England	52737 (13.1)	50564 (13.9)	2173 (5.4)
	Middle Atlantic	35941 (8.9)	32196 (8.9)	3745 (9.3)
	East North Central	39584 (9.8)	35043 (9.7)	4541 (11.3)
	West North Central	64297 (16.0)	61865 (17.0)	2432 (6.1)
	South Atlantic	65356 (16.2)	51419 (14.2)	13937 (34.8)
	East South Central	33305 (8.3)	26409 (7.3)	6896 (17.2)
	West South Central	25730 (6.4)	21209 (5.8)	4521 (11.3)
	Mountain	53570 (13.3)	52813 (14.6)	757 (1.9)
	Pacific	32450 (8.1)	31379 (8.6)	1071 (2.7)

Abbreviation: SD, standard deviation.

**FIGURE 1:**
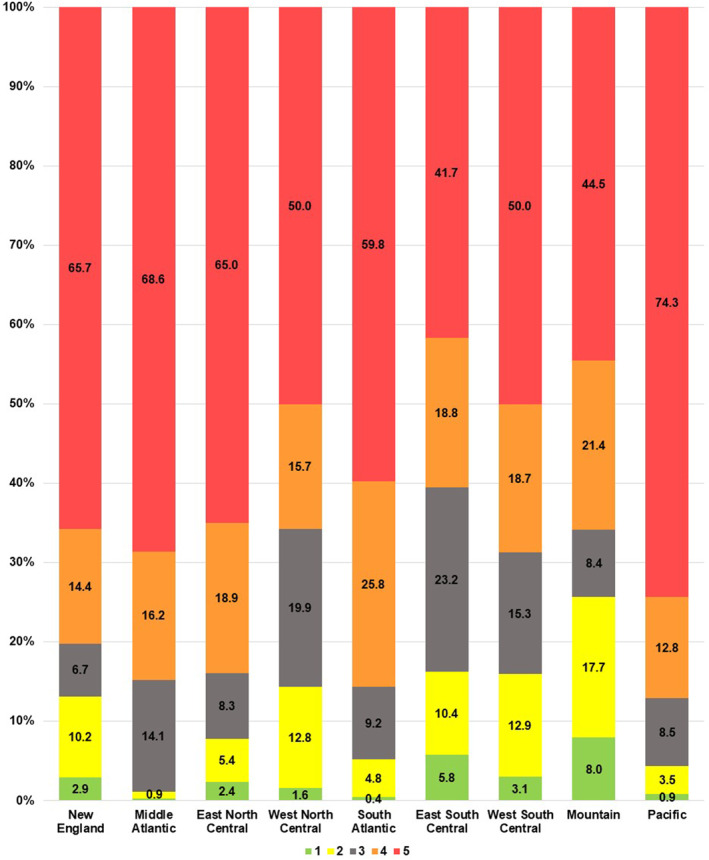
Percentage of respondents within each US Census division from each of the Index of Relative Rurality quintiles.

The distribution of respondents by IRR quintile varied by US Census division (Figure 1). The percentage of respondents residing in the most urban quintile (Q5) ranged from 41.7% in the East South Central division to 74.3% in the Pacific division. The percentage living in the most rural quintile (Q1) ranged from 0.2% in the Middle Atlantic division to 8.0% in the Mountain division.

**FIGURE 2:**
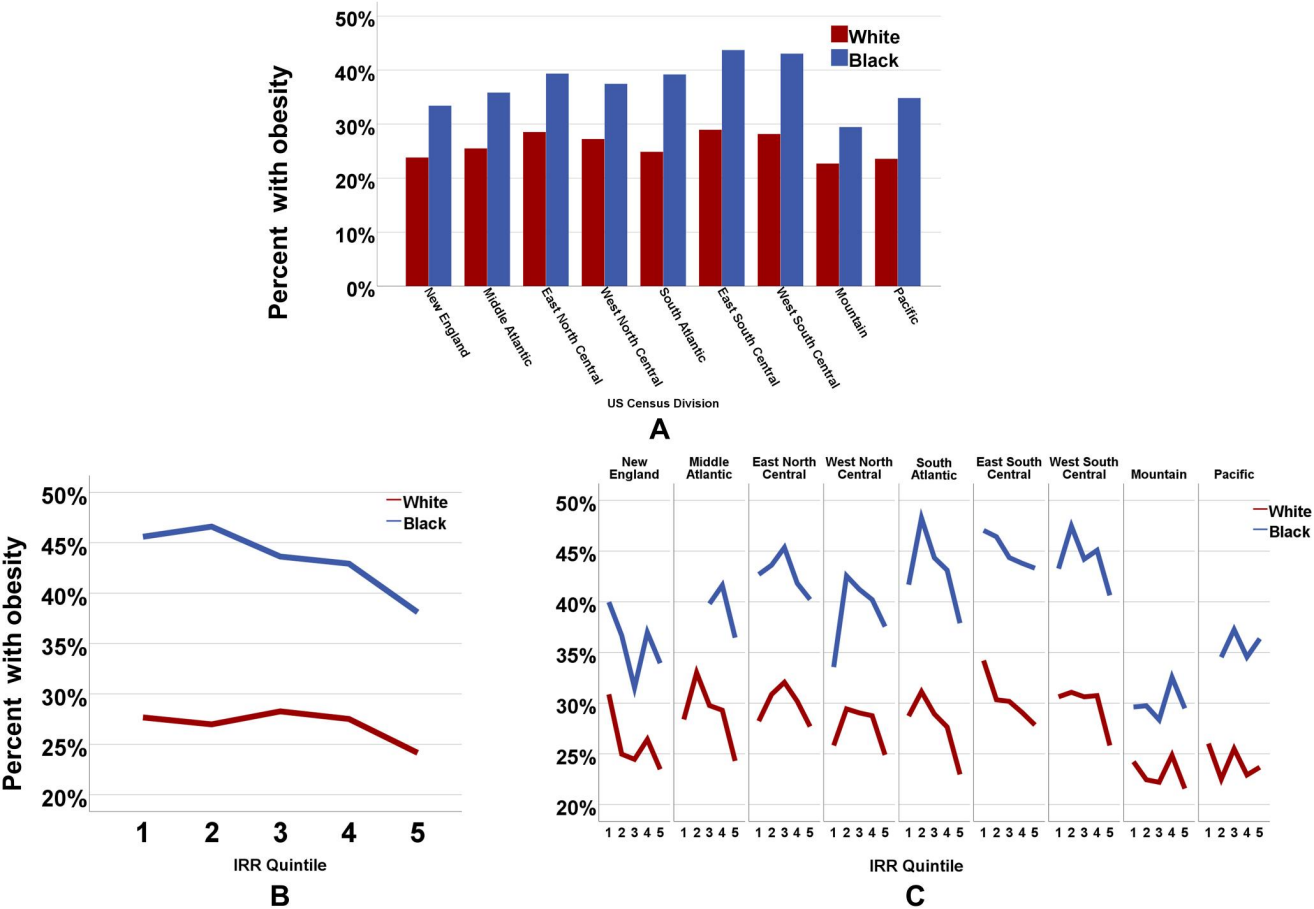
Prevalence of obesity by race (Black and White) by US Census division (Panel A), by Index of Relative Rurality quintile (Panel B), and jointly by US Census division and Index of Relative Rurality quintile (Panel C).

Weighted percentages of obesity by race, US Census division, and IRR quintile are shown in Figure 2. For each division and level of rural‐urban status, Black respondents consistently had higher percentages of having obesity compared to their White counterparts (Panel A). However, respondents living in the Mountain division tended to have the lowest rates of obesity and the smallest Black‐White differences, regardless of the IRR quintile. Overall, the percentage of respondents having obesity increased with increasing rurality among both Black and White respondents (Panel B). Adjusted odds ratios (OR) of obesity within each IRR quintile compared to Whites in the most urban quintile (Q5) are shown in Supplemental Figure S1. The association between obesity and rural‐urban status varied by Census division (Figure 2, Panel C). For example, for both Black and White respondents, there was an upside‐down J‐shaped association between obesity and the IRR quintile for some of the divisions, including the East North Central, West North Central, and the South Atlantic. These trends indicate that the prevalence of obesity was lowest in the most rural and most urban areas, and highest in the areas of intermediate rural‐urban status. However, in the East South Central division, the association was monotonic, indicating that obesity increased with increasing rurality. Furthermore, there was substantial variation in both the overall level of obesity and the magnitude of the Black‐White difference in obesity by both division and IRR quintile.

The weighted and adjusted OR comparing Black to White respondents odds of having obesity overall, and jointly by US Census division and IRR quintile are shown in Table 2. For instance, the 1.86 odds ratio for the Pacific division in the most urban IRR quintile (Q5) indicates that among those in the most urban counties living in the Pacific division (California, Oregon, and Washington), Black respondents were 86% more likely to have obesity than their White counterparts, after adjusting for covariates (Supplemental Table S2). Overall, within each US Census division, Black respondents were significantly more likely than White respondents to have obesity. Odds ratios of having obesity comparing Black respondents to White respondents ranged from 1.51 (95% CI 1.49, 1.53) in the Middle Atlantic division to 1.79 (95% CI 1.76, 1.82) in the Pacific division. In all US counties, regardless of division, the strength of the association between race and obesity was lowest (OR 1.41, 95% CI 1.34, 1.48) in the most rural quintile (Q1), and highest in the second‐most rural quintile (Q2) (OR 1.87, 95% CI 1.82, 1.93).

**TABLE 2:** Prevalence of obesity and odds ratios (OR) (with 95% confidence intervals) of obesity comparing Black respondents to White respondents (referent group) by region, overall and by Index of Relative Rurality quintile (Q1‐Q5)^a^.

	Obesity prevalence (%)		Odds ratios of obesity (comparing black to white respondents)						
	Black	White	Overall	Q1	Q2	Q3	Q4	Q5	Linear trend^b^
All	38.4	25.6	1.71 (1.70, 1.72)	1.41 (1.34, 1.48)	1.87 (1.82, 1.93)	1.86 (1.82, 1.90)	1.79 (1.77, 1.82)	1.77 (1.76, 1.78)	R
New England	31.4	23.0	1.61 (1.56, 1.65)	‐‐	0.45 (0.23, 0.90)	0.10 (0.02, 0.42)	0.71 (0.55, 0.92)	1.69 (1.64, 1.75)	U
Middle Atlantic	31.3	24.1	1.51 (1.49, 1.53)	‐‐	‐‐	3.72 (3.12, 4.43)	1.79 (1.63, 1.97)	1.61 (1.58, 1.63)	R
East North Central	37.5	27.9	1.66 (1.64, 1.69)	0.04 (0.02, 0.07)	0.43 (0.34, 0.54)	1.51 (1.22, 1.86)	1.11 (1.04, 1.18)	1.79 (1.77, 1.81)	U
West North Central	34.8	26.5	1.60 (1.56, 1.64)	‐‐	0.88 (0.74, 1.05)	1.72 (1.56, 1.91)	1.48 (1.31, 1.68)	1.73 (1.69, 1.78)	U
South Atlantic	35.3	24.4	1.73 (1.72, 1.75)	0.68 (0.58, 0.80)	2.31 (2.20, 2.43)	3.23 (2.71, 3.85)	1.90 (1.86, 1.95)	1.74 (1.72, 1.76)	R
East South Central	40.3	28.8	1.72 (1.69, 1.74)	1.48 (1.37, 1.60)	1.53 (1.44, 1.63)	1.89 (1.81, 1.96)	1.46 (1.40, 1.52)	1.84 (1.80, 1.88)	NS
West South Central	38.2	26.4	1.76 (1.74, 1.78)	1.16 (1.03, 1.32)	1.66 (1.55, 1.77)	1.78 (1.70, 1.85)	2.20 (2.12, 2.28)	1.86 (1.83, 1.89)	NS
Mountain	30.7	21.7	1.70 (1.65, 1.75)	0.13 (0.04, 0.42)	2.74 (2.06, 3.65)	0.26 (0.17, 0.39)	0.42 (0.35, 0.52)	1.92 (1.86, 1.99)	U
Pacific	35.0	23.7	1.79 (1.76, 1.82)	‐‐	0.10 (0.05, 0.19)	12.9 (10.3, 16.1)	1.02 (0.95, 1.09)	1.86 (1.83, 1.89)	U

*Note*: U: Urban quintiles significantly higher odds ratios than rural quintiles (*p* < 0.05). NS: No significant linear trend by rural‐urban status (*p* ≥ 0.05).

^a^ Q1 indicates the most rural quintile, Q5 indicates the most urban quintile.

^b^ Linear trend: R: Rural quintiles had significantly higher odds ratios than urban quintiles (*p* < 0.05).

Table 2 also indicates whether there was a monotonic association or linear trend between rural‐urban status and the strength of the association between race and obesity within each Census division. Overall, and for the two Census divisions (Middle Atlantic and South Atlantic), the strength of the race‐obesity association was significantly higher in rural areas than in urban areas. However, for five Census divisions (New England, East North Central, West North Central, Mountain, and Pacific), the strength of the race‐obesity association was significantly higher in urban areas than in rural areas. The association was not statistically significant for both the East and West South Central divisions.

## 4 DISCUSSION

The results of this study validate previous findings identifying Black‐White differences in obesity with respect to geography. Study results determined that within all US Census divisions, the differences in the prevalence of obesity between Blacks and Whites were statistically significant and that this difference was fairly consistent, with the difference ranging from 49% in the Middle Atlantic division to 79% in the Pacific Division. These findings are consistent with previous research, showing that not only did the overall levels of having obesity vary by US region, but also the magnitude of differences between Black and White respondents.^73^ The current study found that the prevalence of obesity was lowest in the Northeast region (New England and Middle Atlantic US Census divisions), where White respondents had a higher prevalence of obesity compared to Black respondents. This study also showed that, after adjusting for demographics and other health issues, Black respondents had higher rates of obesity in all other regions.

The present study's findings extend previous research by highlighting both rural‐urban differences in the overall prevalence of having obesity, as well as variability in the magnitude of the Black‐White difference in the prevalence of obesity.^74^ Studies using a dichotomous measure of rural‐urban status found that rural residents were more likely to have obesity than urban residents, after adjusting for demographic and social factors.^10^, ^12^, ^74^ Another study found a similar rural‐urban difference in overall levels of obesity, and determined that health behaviors, such as dietary quality and physical inactivity, were also lower in rural areas compared to more urban areas.^75^

Furthermore, the present study was the first to examine Black‐White differences in obesity by rural‐urban status. Overall, the Black‐White differences in the likelihood of having obesity were significantly larger in rural areas than in urban areas. It should be noted that among the most rural respondents (Q1), the magnitude of the Black‐White difference was actually the smallest of all the IRR quintiles (OR 1.41), indicating a J‐shaped association when examining the entire sample. Previous research suggests that despite a general trend toward worse health outcomes in rural areas, the most rural and remote populations may have a lower risk of obesity^56^ and improved (increased) rates of cancer survivorship^76^ compared to other populations living in less rural areas. The present study also found this J‐shaped association between rural‐urban status and obesity prevalence as well as the magnitude of Black‐White differences in obesity.

The present study's main contribution is to illustrate critical geographic and regional differences in the associations between rural‐urban status and the magnitude of the Black‐White difference in obesity. In five of the nine US Census divisions—New England, East North Central, West North Central, Mountain, and Pacific—the magnitude of Black‐White differences in obesity was significantly larger in urban areas than in rural areas. However, the association was reversed when examining the sample as a whole and in the Middle Atlantic and South Atlantic divisions. Here, the magnitude of Black‐White differences in obesity was significantly larger in rural areas than in urban areas. There is considerable previous literature indicating regional differences in the US for various health outcomes. These include COVID‐19 cases and deaths,^77^ cardiovascular disease,^78^, ^79^, ^80^ cancer,^81^, ^82^ drug overdoses,^83^ and general health.^84^ One study examined four diseases—cancer, stroke, cardiovascular disease, and chronic obstructive pulmonary disease (COPD)—and found that the factors that predicted the overall level of disease varied substantially by region.^85^

Beyond obesity, a more limited set of studies has examined variability in the magnitude of Black‐White differences by geography for other health outcomes, such as pre‐term birth rates^86^ and coronary heart disease (CHD).^‎90^ Although Black women experienced higher levels of pre‐term birth, there was substantial variability in rates based on both place of residence (US region) and the type of community (rural vs. urban). There were notable regional differences, as well: the magnitude of the Black‐White difference in pre‐term birth rates was significantly higher in the Northeast, South, and Midwest regions compared to the West.^86^ In a study examining temporal changes in mortality from CHD by US division,^90^ CHD mortality decreased in all divisions for both Black and White populations. However, the temporal rate of decline was substantially faster for White populations than for Black populations in four US divisions (Pacific, Mid‐Atlantic, East North Central, and West North Central). The approach and findings of the present study expand upon these studies and highlight important and highly nuanced geographic differences in the prevalence of obesity by race that would otherwise be masked if assessing those racial differences for the country as a whole.

There are numerous potential explanations for why these observed associations and differences occurred. One such explanation is regional differences in the manifestation of structural racism across the country. By definition, structural racism is based on system‐wide, large‐scale cultural, socioeconomic, and political forces that result in health inequities.^27^, ^29^ However, the manifestation of structural racism and how populations experience it with respect to health outcomes may vary regionally.^87^ It could be inferred that racism in the regions in which obesity disparities were highest in rural areas (e.g., Middle Atlantic and South Atlantic) may be more pronounced in rural areas than in urban areas. These factors may, in turn, create more obesogenic environments in rural areas, particularly for Black populations, which may experience greater levels of stress and lower levels of security due to those long‐term cultural, social, and political factors.^88^ In other areas, such as New England, racism may be more strongly experienced in urban areas, particularly in historically Black communities, which may have been neglected through decades of harmful policies^33^ and cultural factors, such as “White flight” from urban areas and de facto segregation.^89^ More research is needed to identify and address the root causes of these stark geographic differences in Black‐White disparities in obesity by rural‐urban status.

The findings of the present study should be interpreted in the context of several important limitations. First, as the data were cross‐sectional, causation cannot be assessed. Second, the sample had a greater proportion of respondents from the most urban quintiles of US counties, which limited statistical power in the most rural counties (Q1). Third is the “modifiable area unit problem”, which identifies critical spatial variability with respect to the size and physical layout of counties across the country, which can result in statistical bias of model‐based estimates.^90^, ^91^ Fourth, the level of spatial aggregation for rural‐urban status was done at the county level. Previous research suggests that geographic associations may vary by the level of spatial aggregation utilized.^92^, ^93^ Relatedly, the analysis did not account for potential spatial autocorrelation because not all counties were represented in the BRFSS sample. Next, rural‐urban status is a complex and multi‐dimensional characteristic.^94^ Although the measure used to characterize rural‐urban status, the IRR,^64^ considers four aspects of rural‐urban status and has been validated in prior studies,^66^, ^67^, ^68^, ^69^, ^70^, ^71^ there may be other factors associated with rurality that were not included in this measure. Furthermore, analyses were restricted to non‐Hispanics to reduce the potential for bias stemming from self‐reports and misclassification of Hispanic ethnicity,^90^ so the findings cannot be extended to those identifying as Hispanic. Another consideration is that the data were from 2012, and obesity rates have likely increased since the data were collected, in addition to other changes in SDH during that period. Data from 2012 were used because it was the most recent year in which the BRFSS included county‐level geographic identifiers, which were used to spatially link each subject to Census data used in the study. Lastly, only a limited set of confounders were included in the multivariable analysis, and the associations between the confounders and the main exposure variable were evaluated in the entire sample. This approach leads to two limitations: residual confounding and the potential for the associations between each of the confounders and the main exposure variable (race) to vary by region.

Despite these limitations, the study has a number of notable strengths. First, the study is the first to evaluate the magnitude of Black‐White differences in having obesity individually and jointly by rural‐urban status and geographic region using a large, nationally representative dataset. The observed geographic variability in the associations between rural‐urban status and Black‐White differences in obesity underscores the critical need to consider the geographic context when creating and implementing health policies and programs designed to reduce such differences and promote health equity. To maximize the effectiveness of any such policies and programs to reduce disparities and promote health equity, the specific, area‐level causes and contextual factors of such disparities must be understood and addressed. Those causes and contextual factors may differ across regions, so one‐size‐fits‐all approaches may be limited in effectiveness.

Another important strength is the assessment of the association between rural‐urban status and obesity across US divisions using rural‐urban status as an ordinal, five‐level variable and considering both monotonic and non‐monotonic associations. There is increasing recognition that population health studies should move beyond dichotomous measures of rural‐urban status toward seeing rural‐urban status as a continuum.^94^, ^95^, ^96^, ^97^ Results of the present study suggest that although there may be overall trends toward better health outcomes in rural or urban areas, depending upon the region, many such associations were muted in the most rural and remote areas, resulting in a J‐shaped association. More research is needed to validate these findings and determine what potentially modifiable factors contribute to these non‐monotonic associations.

Overall, the study findings corroborate the vast majority of previous literature showing that Black‐White differences in obesity are pervasive and persistent across geographies. However, the magnitude of those differences and the overall prevalence of obesity varied substantially by both rural‐urban status and region. Additional research is needed to better understand the community‐level drivers of these associations at the local level and provide a more comprehensive understanding of why these differences occur. Mixed methods approaches using qualitative information from key community stakeholders could provide insights into critical cultural, socioeconomic, and environmental factors that may vary from place to place. Findings from such research could uncover highly influential local attributes that may help explain variability in these findings on a finer geographic scale.

Creating effective policies, programs, and interventions needs to consider the community factors such as culture, social determinants, and environmental factors that give rise to these complex patterns of health inequities. The results of this research underscore the notion that the community‐based factors that promote or impede health and health disparities in obesity likely differ from place to place. Effective measures to target those inequities may require a deeper, systematic understanding and addressing of these micro‐level factors that likely vary by geography. For example, efforts to create healthier, less obesogenic built environments by promoting physical activity (e.g., parks, green space, sidewalks, etc.) should consider other factors that could inhibit physical activity, such as crime rates or perceived safety, or traffic patterns,^98^ which themselves may vary by neighborhood or even by block.^99^ The results of this study suggest that efforts to reduce obesity and obesity‐related health disparities that may work well in one area may not necessarily work in another seemingly similar area due to these underlying factors. Such broad‐brush, “one‐size‐fits‐all” efforts may be strengthened by understanding the unique needs of different populations in diverse settings across the country and adapting those efforts to meet those needs of populations at the local level.

## CONFLICT OF INTEREST STATEMENT

The authors declare no conflicts of interest.

## Supporting information

Supporting Information S1
