# Supplementary material for: Geographic differences in the magnitude of black‐white disparities in having obesity
Source: Obes Sci Pract. 2023 May 26;9(5):516–28. doi: 10.1002/osp4.679 (PMC10551120; doi:10.1002/osp4.679)
Supplement: Supplementary file 1 — Supporting Information S1 [file OSP4-9-516-s001.docx]

**SUPPLEMENTAL MATERIAL**

**Supplemental Table 1: List of states by US Census division**

| New England | Connecticut, Maine, Maassachusetts, New Hampshite, Rhode Island, Vermont |
| --- | --- |
| Middle Atlantic | New Jersey, New York, Pennsylvania |
| East North Central | Indiana, Illinois, Michigan, Ohio, Wisconsin |
| West North Central | Iowa, Kansas, Minnesota, Missouri, Nebraska, North Dakota, South Dakota |
| South Atlantic | Delaware, District of Columnia, Florida, Georgia, Maryland, North Carolina, South Carolina, Virginia, West Virginia |
| East South Central | Alabama, Kentucky, Mississippi, Tennessee |
| West South Central | Arkansas, Louisiana, Oklahoma, Texas |
| Mountain | Arizona, Colorado, Idaho, New Mexico, Montana, Utah, Nevada, Wyoming |
| Pacific | Alaska, California, Hawaii, Oregon, Washington |

**Supplemental Table 2: Adjusted odds ratios (and 95% confidence intervals) for propensity score matching analysis**

|  | Odds ratio | 95% confidence interval |
| --- | --- | --- |
| Variable |  |  |
| Age (years) | 0.98 | (0.98, 0.98) |
| Annual income ($) |  |  |
| < 25,000 (ref) | 1 |  |
| 25,000-49,999 | 0.63 | (0.61, 0.65) |
| > 50,000 | 0.40 | (0.38, 0.42) |
| Missing | 0.61 | (0.59, 0.63) |
| Currently employed |  |  |
| No (ref) | 1 |  |
| Yes | 0.94 | (0.92, 0.97) |
| Education |  |  |
| Less than college (ref) | 1 |  |
| Bachelor's or higher | 0.74 | (0.72, 0.76) |
| Currently married |  |  |
| No (ref) | 1 |  |
| Yes | 0.48 | (0.47, 0.49) |
| Current smoker |  |  |
| No (ref) | 1 |  |
| Yes | 0.80 | (0.78, 0.82) |

**Supplemental Figure: Adjusted odds ratios (with 95% confidence intervals) of obesity by race (Black/White) and IRR quintile compared to Whites in the most urban quintile (Q5)**
